# Neuro-Analogical Gate Tuning of Trajectory Data Fusion for a Mecanum-Wheeled Special Needs Chair

**DOI:** 10.1371/journal.pone.0169036

**Published:** 2017-01-03

**Authors:** Ahmed K. El-Shenawy, M. A. ElSaharty, Ezz Eldin zakzouk

**Affiliations:** Arab Academy for Science, Technology and Maritime Transport, Electric and Control Department, College of Engineering and Technology, Alexandria, Egypt; West Virginia University, UNITED STATES

## Abstract

Trajectory tracking of mobile wheeled chairs using internal shaft encoder and inertia measurement unit(IMU), exhibits several complications and accumulated errors in the tracking process due to wheel slippage, offset drift and integration approximations. These errors can be realized when comparing localization results from such sensors with a camera tracking system. In long trajectory tracking, such errors can accumulate and result in significant deviations which make data from these sensors unreliable for tracking. Meanwhile the utilization of an external camera tracking system is not always a feasible solution depending on the implementation environment. This paper presents a novel sensor fusion method that combines the measurements of internal sensors to accurately predict the location of the wheeled chair in an environment. The method introduces a new analogical OR gate structured with tuned parameters using multi-layer feedforward neural network denoted as “Neuro-Analogical Gate” (NAG). The resulting system minimize any deviation error caused by the sensors, thus accurately tracking the wheeled chair location without the requirement of an external camera tracking system. The fusion methodology has been tested with a prototype Mecanum wheel-based chair, and significant improvement over tracking response, error and performance has been observed.

## Introduction

For decades, the medical care community has been working on developing a smart environment for special needs and elderly patients [1] [2] and [3]. The autonomous wheel chair is the main element in such an environment which requires highly navigational performance to guarantee efficient integration in any smart environment. Till now, the commercial wheeled chair is partially autonomous to compensate for the user’s physical deficiencies. The user always collides with the problem of local navigation. Therefore, the chair must have the properties of localization, path planning and position update. These themes represent a new trend for the robotics community in the past few years [4] [5] [6]. In [7], wheeled chair is integrated with a framework to estimate the intention of the user. It determines whether the user needs assistance to achieve his/her intention. A deictic approach is used in [8] for driving assistance smart wheelchair between doors and passages. All navigation systems are easier to develop with a predefined map of the working environment. In some environments the reconstruction and maintenance of the maps is needed [9]. The problem of position update is very important, as the whole navigational system depends on calculating accurate position coordinates for the chair. Operating in a wireless environment improves the performance of such chair where the detection of accidents and approximate tracking of a user is efficient [10]. Further more, the efficient routing is one of the current major challenges in the area of wireless sensor networks. Where reducing energy consumption and increasing the network life time is considered [11] [12] [13]. The camera is considered as one of the main sensors to deliver accurate information from environments. For example, it is used to combined a vision-based posture classification scheme to extract further information about the user when an alert occurs [10]. Kalman filters are one of the main common methods for sensor fusion, although it is more likely used to filter and fuse parallel images to compare parallel and converged cameras [14]. However, the availability to use a wireless network and a camera vision sensor is not applicable in many environments. Therefore, the wheel chair should be more dependable on its internal sensors only for its position update. Calculating the accurate position of a wheeled mobile base (chair) may be achieved by using external sensors, internal sensors or a combination of both. Normally, the wheel chair depends mainly on its internal sensors attached to the platform.

However, the internal sensors are affected by the chair’s environmental temperature, humidity, and slippage. For example, the shaft encoders do not detect wheel slippages, and the inertia motion unit (IMU) always has a shifting offset depending on a temperature and humidity. Such offset will create an accumulated error when passed through an integration operation for velocity or acceleration detection. Several approaches have been presented in the literature to minimize and eliminate the effect of this offset [15]. However, such methods are complicated and are not always reliable. On the other hand, the camera tracking system does not have such an disadvantage. Therefore, it is used within the wheel chair control structure.

In certain environments the camera tracking system may not be available or feasible. In this paper, a method is developed to fuse the measurements from internal sensors (IMU and shaft encoder) to achieve accurate position coordinates of the wheeled chair which can yield an accuracy similar to a camera tracking system. This is performed by combining the mean of neural networks and analogical gates to fuse the internal sensors position coordinates.

The work presented in this part of a project funded by the Arab Academy for Science, Technology and Maritime transport. The motivation of the project is to create a “Smart Environment for Elderly and Disabled Users” (SEED). The wheel chair is the main element of this environment, which must be flexible in maneuvering and assisting the user in navigation through his/her home. Flexibility in maneuvering is preliminarily achieved through the use of mecanum wheels attached to the wheeled mobile base platform to increase the degree of freedom (DOF) of motion.

The paper starts with demonstrating the chair configuration in section (1). The navigation system is explained in section (2). Section (3) defines Analogical gates and its properties, especially the OR analogical gate. The integration of the neural networks with the analogical gates is presented in section (4). Finally, the proposed method performance is illustrated in section (5) with experimental results.

## 1 Wheel Chair Configuration

Having a chair with a flexible motion is one of the main objectives. Therefore the chair must have motion holonomic properties, which implies that the number of robot velocity DOF are equal to the number of position coordinates. A rigid body has six DOFs [16], represented by displacement axes *X*, *Y* and *Z* and the rotational angles *θ*_*x*_, *θ*_*y*_ and *θ*_*z*_, A holonomic wheeled mobile robots (WMR) is a robot that can drive in three DOF (*X*, *Y* and *θ*_*z*_ or Φ). A non-holonomic WMR is a the robot that cannot perform the 3DOF mobility. Where the non-holonomic WMR cannot move sideways in the direction of the *X* axis with respect to the platform co-ordinates [17].

**Fig 1:**
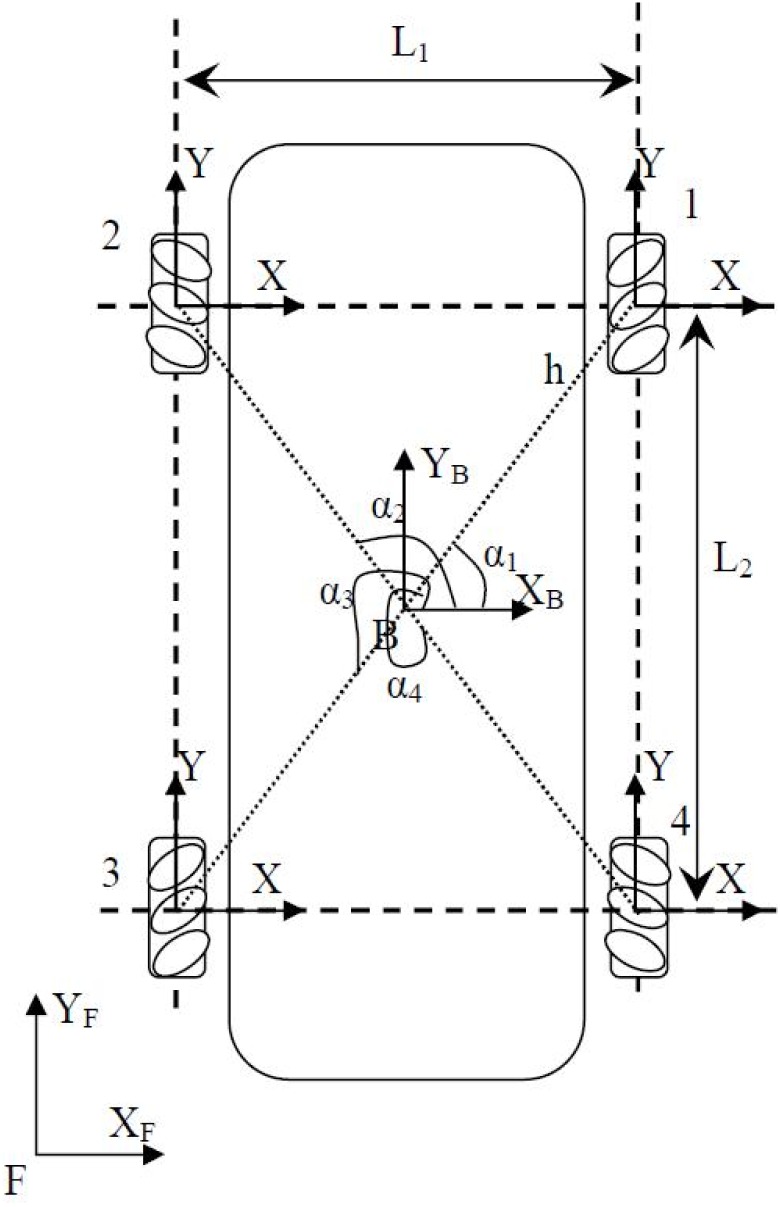
The Wheeled Chair configuration.

The wheel platforms with holonomic properties have been investigated in several studies [17] [18] and with different configurations [19]. The mecanum wheel is one of the most recommended wheels for holonomic mobility properties. Therefore, it is used as a wheeled base for the wheel chair. The platform configuration used is shown in Fig 1; it has been studied before by several researchers [20] [21] and [22].

**Fig 2:**
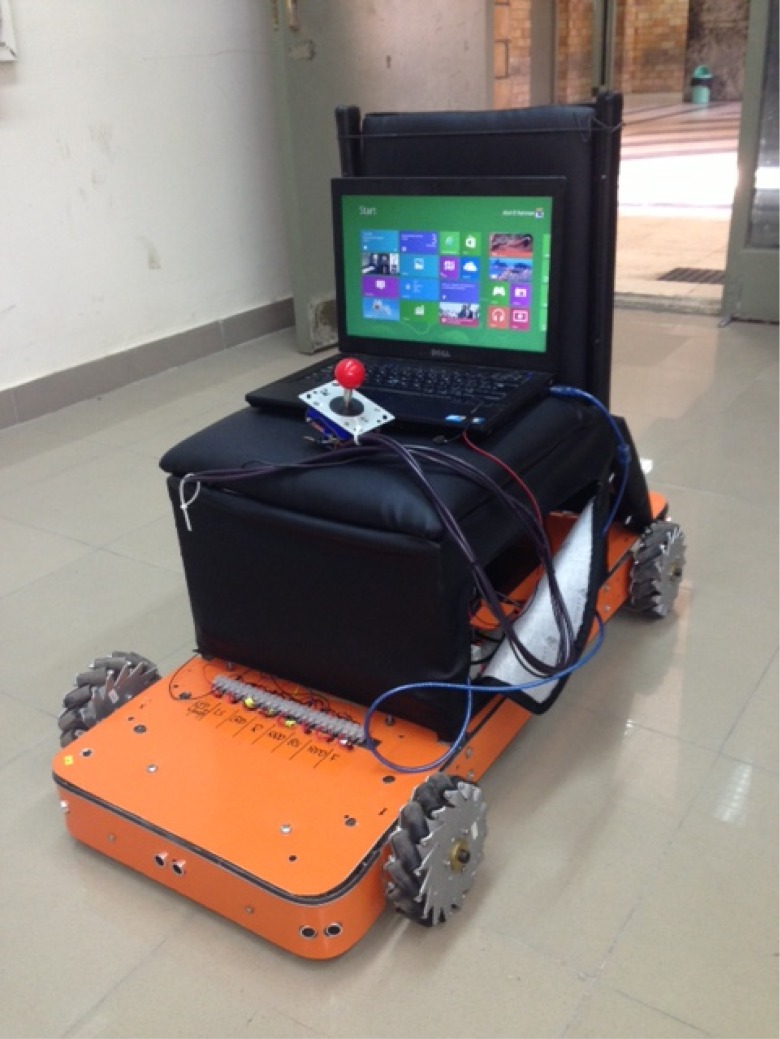
The Chair Real-Time Setup.

The hardware setup used in this paper is V.01 of the wheeled chair shown in Fig 2. The chair consists of two main parts: 1) the wheeled platform configuration and 2) the electric hardware setup. The platform has a rectangular shape supported by four Mecanum wheels. Each wheel is capable of supporting a maximum weight of 15 Kg with a radius of 65 mm. The mecanum wheel used in the configuration consists of nine rollers made from delrin. The chair platform is attached to wheels of +45° rollers and wheels with −45° rollers on each side.

The platform is equipped with four DC motors to actuate the wheel’s angular velocity. The motors operate at 12V DC with a rated speed of 250rpm and rated power of 41 watts. Each motor has planetary steel gear box and an incremental encoder attached to its shaft. The motors are directed by a high power DC motor drivers, which can drive up to 10A continuously and 15A peak current for 10 seconds. Eight ultrasonic sensors are used for distant objects calculations. They are used to assess the collision avoidance behavior. Furthermore, a gyro sensor is used as well for rotational acceleration measurements and for the platform motion control. The main control unit is based on an Arduino Mega 2560 R3.

The omni-directional capabilities of the platform depend on firm contact with the surface. The platform’s parameters are described in Table 1.

**Table 1:** The platform parameters.

Platform Parameters	Value	Units
*L*_1_	0.6	*m*
*L*_2_	1.04	*m*
*h*	0.30	*m*
*R*	0.075	*m*
*r*_0_	0.20	*m*
*M*_*p*_ (*P*_*l*_ mass)	22	*Kg*
*I*_*p*_ (*P*_*l*_ inertia)	3.51	*Kg* *m*^2^

## 2 Navigation System

**Fig 3:**
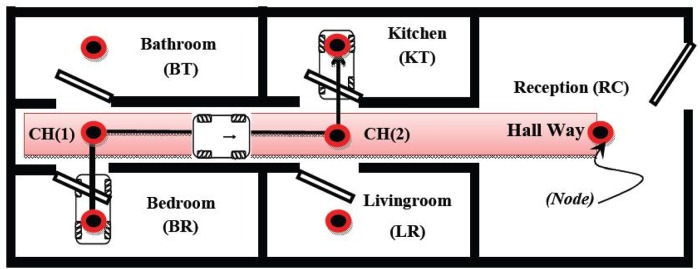
The apartment node map.

It is assumed that the chair will navigate with a known map and knowledge about its local environment, for example, a user’s apartment. The apartment consists of seven main nodes as follows: 1) bedroom (BR), 2) living-room (LR), 3) bathroom (BT), 4) kitchen (KT), 5) reception (RT) and 6) hallway (CH1) and (CH2), as shown in Fig 3. These are the basic rooms in a typical apartment, however there can be fewer rooms. The Hallway (CH1) is considered as the reference point. The chair should reach first in order to drive from one room to another.

The navigation system depends mainly on motion control from one node to another and on the position control loop that drives between each two nodes. First, the bedroom (BR) is considered as the base node with coordinates (*X*, *Y*) = (0, 0), and the other node’s coordinates will be the displacements from that node.

**Fig 4:**
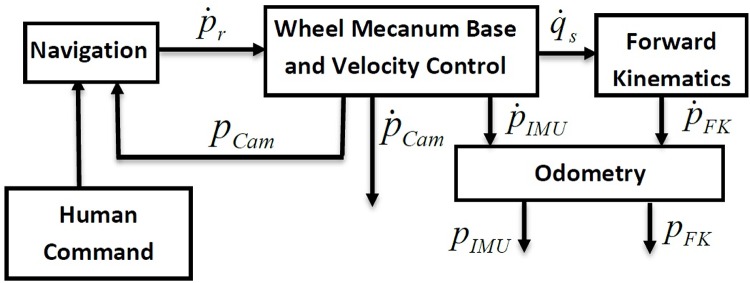
The Wheeled Chair Control System.

The control system of wheeled chair is shown in Fig 4. The chair is assumed to be controlled by voice commands and joystick commands. First, some symbols will be defined to illustrate the control system and the proposed work:

*P* position coordinates in *X*, *Y*, Φ
$\dot{P}$ velocity coordinates in $\dot{X},\dot{Y},\dot{\Phi }$
$\ddot{P}$ acceleration coordinates in $\ddot{X},\ddot{Y},\ddot{\Phi }$
${\dot{P}}_{Cam}$ velocity coordinates delivered from the camera tracking system
*P*_*Cam*_ Position coordinates delivered from the camera tracking system
${\dot{P}}_{IMU}$ velocity coordinates delivered from the IMU system
*P*_*IMU*_ Position coordinates delivered from the IMU system
${\dot{P}}_{FK}$ velocity coordinates delivered from the forward kinematics
*P*_*FK*_ Position coordinates delivered from the forward kinematics
${\dot{P}}_{r}$ Reference velocity coordinates
${\dot{q}}_{s}$ Angular wheels velocity ${\dot{\theta }}_{x_{1}},{\dot{\theta }}_{x_{2}},{\dot{\theta }}_{x_{3}},{\dot{\theta }}_{x_{4}}$

The robot coordinates are measured and estimated by three main methods: 1) camera tracking, 2) IMU and 3) forward kinematics estimation. Firstly, in this case study, velocity (${\dot{P}}_{cam}$) and position coordinates (*P*_*cam*_) generated from the camera tracking system are used within the control system. These data are used because they are the most accurate position coordinates, which will be shown in the experimental data section.

Secondly, the measurements are taken directly from the IMU system locked on the chair. The IMU system normally measures the acceleration of the linear motion ($\ddot{X},\;\ddot{Y}$) and angular acceleration of the angular motion ($\ddot{\Phi }$), using a combination of an accelerometer and gyroscope. By means of integration, the robot velocities (${\dot{P}}_{IMU}$) and position coordinates (*P*_*IMU*_) can be calculated.

Thirdly, the robot velocity reference value ${\dot{P}}_{r}={\left[\begin{array}{ccc} \dot{X} & \dot{Y} & \dot{\Phi } \end{array}\right]}^{{}^{T}}$ is the command signal for the wheel chair control system. Shaft encoders on the wheel deliver the wheels angular velocities: ${\dot{q}}_{s}={\left[\begin{array}{cccc} {\dot{\theta }}_{x_{1}} & {\dot{\theta }}_{x_{2}} & {\dot{\theta }}_{x_{3}} & {\dot{\theta }}_{x_{4}} \end{array}\right]}^{{}^{T}}$. The wheels’ angular velocities are transformed to chair co-ordinates ${\dot{P}}_{FK}={\left[\begin{array}{ccc} \dot{X} & \dot{Y} & \dot{\Phi } \end{array}\right]}^{{}^{T}}$ by means of the forward kinematics solution, which is described in [23] using the following model:

$$\begin{array}{c} \dot{p}=J_{f}{\dot{q}}_{s}, \end{array}$$ (1)

where, ${\dot{q}}_{s}$ is the sensed wheel velocities, *J*_*f*_ is the forward kinematic solution, and $\dot{p}$ is the desired platform velocities. The actuated inverse solution is

$$\begin{array}{c} J_{f}=\frac{R}{4}\left[\begin{array}{cccc} 1 & -1 & 1 & -1\\ 1 & 1 & 1 & 1\\ -\frac{\sqrt{3}-1}{h(\sqrt{3}-2)} & \frac{\sqrt{3}-1}{h(\sqrt{3}-2)} & \frac{\sqrt{3}-1}{h(\sqrt{3}-2)} & -\frac{\sqrt{3}-1}{h(\sqrt{3}-2)} \end{array}\right], \end{array}$$ (2)

where *R* is the wheel radius and *h* is the distance from each wheel to the robot coordinates. The wheels encoders measure the angular velocity of each wheel individually: ${\dot{q}}_{s}={\left[\begin{array}{cccc} {\dot{\theta }}_{x_{1}} & {\dot{\theta }}_{x_{2}} & {\dot{\theta }}_{x_{3}} & {\dot{\theta }}_{x_{4}} \end{array}\right]}^{{}^{T}}$.

The difference between the three systems may be elaborated using the following experiment. As mentioned before, the wheel chair is tested within a known environment and its navigational systems is proven in [23]. The node coordinates of the apartment shown in Table 2.

**Table 2:** Navigational node coordinates.

Node	(X[m], Y[m]) = index	Node	(X[m], Y[m]) = index
BR	(0[m], 0[m]) = 1	LR	(1.9[m], 0[m]) = 4
BT	(0[m], 2.5[m]) = 2	KT	(1.9[m], 1.3[m]) = 3
CH1	(0[m], 1.3[m]) = 5	RC	(3.2[m], 1.3[m]) = 7
CH2	(1.9[m], 1.3[m]) = 6		

**Fig 5:**
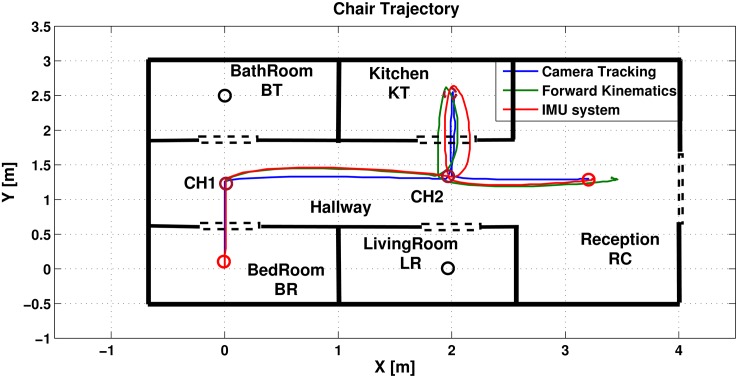
Wheel hair trajectories for FR, IMU and camera positioning systems.

The experiment demonstrates the trajectories of three different position update systems (forward kinematics, IMU and camera tracking systems). Commands given to the system are as follows: first, reaching Kitchen from bedroom (BD → KT), secondly, drive to the reception from kitchen (KT → RC). The control system proposed in [23] generated the following sequence of indexed nodes 1, 5, 6, 3 and 7. Position control system was used to drive the chair from one node to another according to its initial and goal co-ordinates on the (X, Y) axes, while the rotational angle had a reference value of Φ = 0°. The chair trajectory in Fig 5 shows that the proposed system fulfilled the sequence.

**Fig 6:**
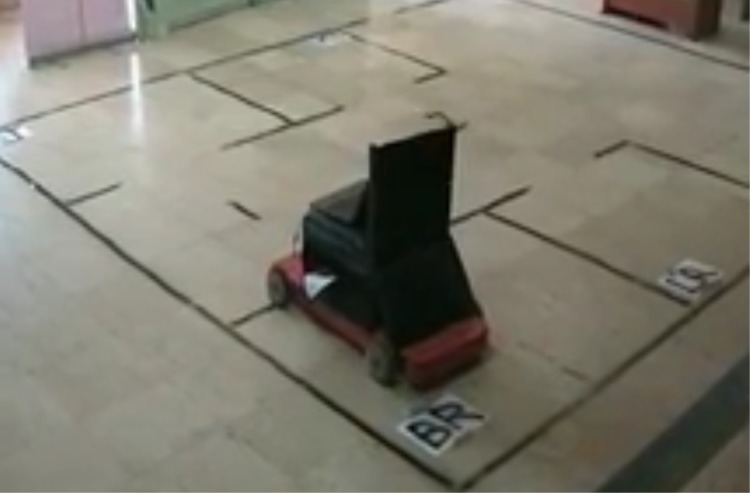
Driving from BR to Node1.

**Fig 7:**
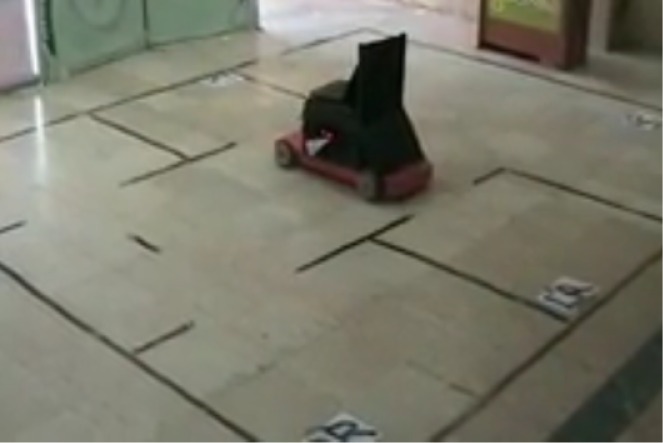
Driving from Node 2 to KT.

Figs 6 and 7 shows the small environment used to test the navigational systems. Fig 6 represents the first motion to drive from bedroom to CH1, while Fig 7 shows the motion from CH2 to the kitchen.

The trajectories of the three positioning systems are shown in Fig 5. It is quite clear that the camera tracking system gives the most exact position coordinates, while the others have clearly noticeable errors. To evaluate the performance of the IMU and forward kinematics positioning systems, their resulted trajectories will be compared to the camera positioning system. The error between each trajectory will be calculated referred to the camera trajectory as follows:

$$\begin{array}{c} E_{FK}=\sqrt{{\left(X_{Cam}-X_{FK}\right)}^{2}+{\left(Y_{Cam}-Y_{FK}\right)}^{2}} \end{array}$$ (3)

for the FK positioning system and for the IMU system:

$$\begin{array}{c} E_{IMU}=\sqrt{{\left(X_{Cam}-X_{IMU}\right)}^{2}+{\left(Y_{Cam}-Y_{IMU}\right)}^{2}}. \end{array}$$ (4)

The mean error equations $\frac{1}{n}\sum_{i=1}^{n}E_{FK}\left(i\right)$ and $\frac{1}{n}\sum_{i=1}^{n}E_{IMU}\left(i\right)$ are used to show how each trajectory is close to the camera (where n is the number of points taken on the trajectory). The forward kinematics (FK) resulted in a (0.381[m]) mean error, while the IMU system resulted in a (0.258 [m]). Since the apartment dimensions are scaled with a ratio of 1:5, therefore, the errors are rescaled with the same ratio to be (1.9[m]) for the FK, and (1.3[m]) for the IMU system.

Theses errors are not acceptable by the control system, which is an important reason for adding the camera tracking system to the chair control system. Alternatively, the next sections will propose a novel fusion system depending on the integration between analogical gates theory and the neural networks algorithms, what we call a “neuro-analogical gate” (NAG).

## 3 Analogical Gates

**Fig 8:**
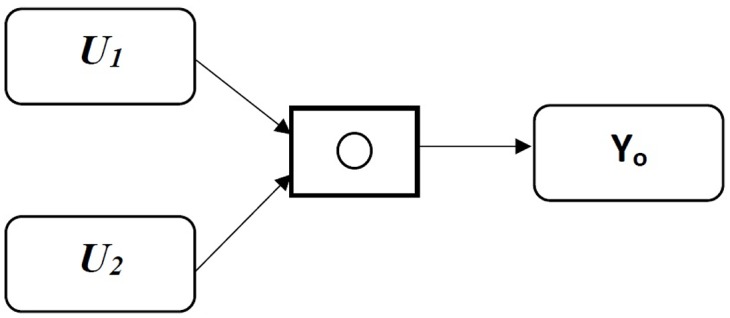
Analogical Gate.

The analogical gates are divided into two types: symmetric and asymmetric. The symmetric gates perform an operation similar to their logic counter, such as AND, OR and XOR. In this work, the structure of an OR gate is used; however some of its features will be changed according to the proposed neurological system. The gate will combine a coordinates system on the X and Y axes. These data are combined pair-wise by a binary operation as shown in Fig 8

This means that if *o* denotes a binary operation then:

*y*_*o*_ = *u*_1_ *o* *u*_2_ always exist = = > *o* is well defined

*u*_1_, *u*_2_, *y*_*o*_ ∈ *V*, *V* = {*v*|*v* = [*v*_*min*_, *v*_*max*_], *v* ∈ ℜ} = = >*V* is closed under *o*

*y*_*o*_ = *u*_1_ *o* *u*_2_ is unique on *U*_1_ × *U*_2_ ∀*u*_2_ ∈ *U*_1_, *u*_2_ ∈ *U*_2_, *U*_1_, *U*_2_ ⊆ *V*

**Fig 9:**
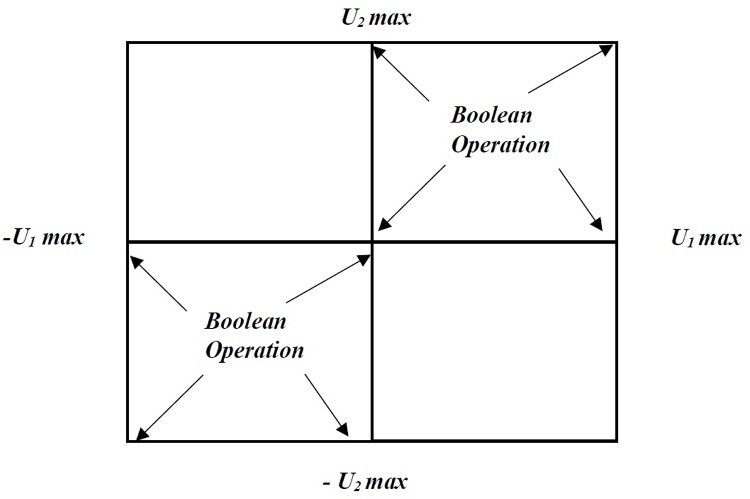
Input Structure.

The analogical gates borrow their names from the analogy to Boolean logic gates on the vertices of first and third quadrant in the input space, as shown in Fig 9.

An analogical gate is presented by the binary relation

**Fig 10:**
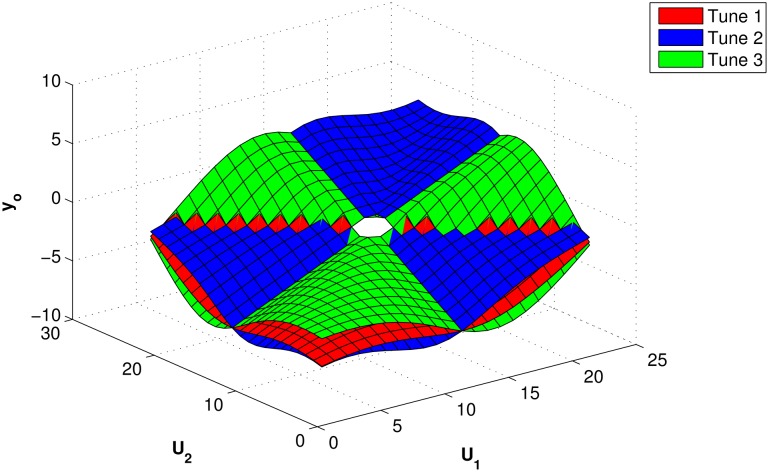
Surface representing analogical OR gate for different tuning parameter values.

*y*_*o*_ = *u*_1_ *o* *u*_2_

on the behavior element *u*_1_ and *u*_2_. Further, for the definition of the analogical gates, we use the exponential function Eq (5) as stated in [24].

$$\begin{array}{c} \xi \left(u_{1},u_{2}\right)=e^{-}\left(\frac{a{u_{1}}^{2}+bu_{1}u_{2}}{{u_{1}}^{2}+{u_{2}}^{2}}\right) \end{array}$$ (5)

and *u*_1_, *u*_2_ ∈ ℜ. Consider the following formulation for OR gate

$$\begin{array}{c} z=u_{1}\;⊕u_{2}=u_{1}\xi \left(u_{2},u_{1}\right)+u_{2}\xi \left(u_{1},u_{2}\right) \end{array}$$ (6)

and according to [24], *a* = 1.028 and *b* = 0.357, so the formula satisfy the OR conditions. However, any changes in *a* and *b* results different the system behavior, as shown in Fig 10. The figure shows the surface horizon for the OR gate with three different *a* and *b* values, as shown in Table 3.

**Table 3:** Values of *a* and *b* related to Fig 10.

	*a*	*b*
Tune 1	1.02	0.357
Tune 2	0.9	−0.35
Tune 3	1.5	1.05

Different values of *a* and *b* changes the properties of the OR gate. The resulting gates surface horizons may be used to fuse different inputs, as shown in Fig 10.

If the fusion is used for FK and IMU trajectories using the position coordinates, there will be an infinitely possible number of coordinates. However, the chair velocities are limited to ±0.2[*m*/*s*] in any direction. Therefore, the fusion considered for the chair velocity levels. The velocity vectors *V*_*FK*_ and *V*_*IMU*_ represent different values for the chair *X* or *Y* velocities.

**Fig 11:**
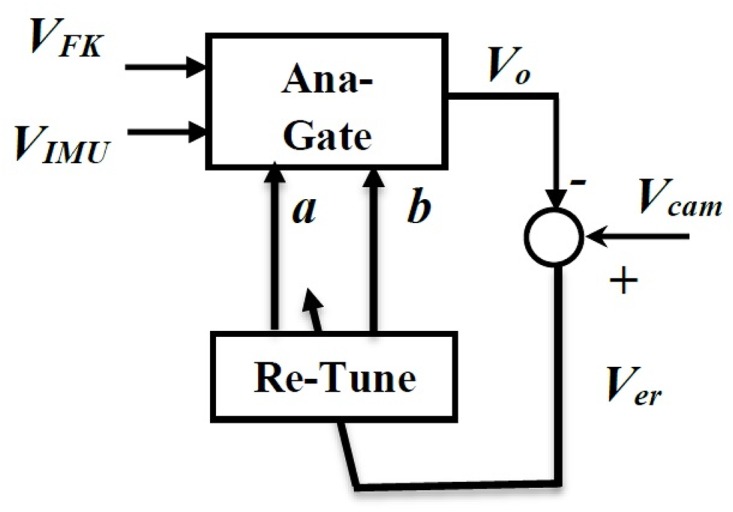
Tuning analogical gate.

The parameters *a* and *b* values are responsible for tuning the gate to meet the values of *V*_*Cam*_. The experiment shown in section (2) includes almost 1200 samples, 120 of them are considered the main operating points to tune their *a* and *b* values as shown in Fig 11. Table 4 shows the some operating points used.

**Table 4:** The platform parameters.

*V*_*FK*_	*V*_*IMU*_	*V*_*cam*_	a	b
0.1900	0.1600	0.0700	1.9830	1.0000
0.2500	0.2300	0.1800	0.9600	1.0000
0.2900	0.2700	0.2800	0.4000	1.0000
0.3200	0.3000	0.3600	0.1145	1.0000
0.3200	0.3400	0.3700	0.1000	1.0300
0.2800	0.3000	0.3100	0.1000	1.1240
0.2600	0.2800	0.2600	0.1000	1.3240
0.2200	0.2600	0.2300	0.1000	1.0700
0.2000	0.2400	0.1900	1.0140	1.0000
−0.2400	−0.2000	−0.2500	0.2000	1.0000
−0.2100	−0.1600	−0.2700	0.2000	0.4520
−0.2300	−0.2400	−0.1700	1.0500	1.0000
−0.1600	−0.1900	−0.0900	1.5000	1.3800
−0.1200	−0.1500	−0.0600	1.6000	2.2000
−0.0200	−0.0100	−0.0200	0.2000	0.9000
−0.0300	−0.0400	0	1.0000	2.5000
−0.0100	0	0	2.0000	2.5000

These operating point are chosen from the different situations found between nodes (5, 6) and (6, 3), representing the horizontal and vertical motion.

The new gate is integrated with neural networks to result the “neuro-analogical gate” (NAG). The variables *a*, *b* of the gate are generated from a feed-forward neural network to fuse the trajectory data using analogical gate.

## 4 Neuro-Tuned Gates

The training process used for the multi-layer feed-forward (MLF) neural network will be supervised, based on the test sets with known inputs and outputs. Each neuron in a particular layers is connected to all neurons in the next layer. The connection between the *i*^*th*^ and the *j*^*th*^ neurons is characterized by the weight coefficient *W*_*ij*_, the *i*^*th*^ neuron in one layer is biased by *ϑ*_*i*_. The output value of the *i*^*th*^ neuron *x*_*i*_ is defined by the function

$$\begin{array}{c} \zeta_{i}=\vartheta_{i}+\sum_{j=1}^{n}W_{ij}x_{j}, \end{array}$$ (7)

where n is the number of neurons in the previous layer and

$$\begin{array}{c} x_{i}=f\left(\zeta_{i}\right), \end{array}$$ (8)

**Fig 12:**
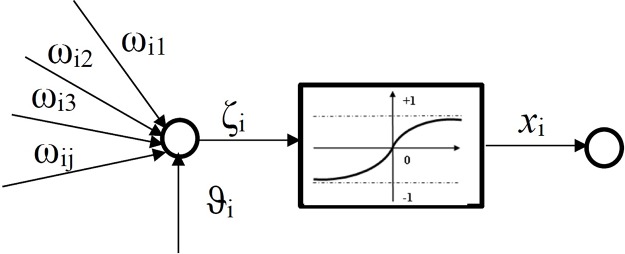
Single-Input Neuron.

*f*(*ζ*_*i*_) is the transfer function carried throughout layer *j*, transferring the signal to the *i*^*th*^ neuron, as shown in Fig 12. The tansigmoid function is used in the proposed network,

$$\begin{array}{c} f\left(\zeta_{i}\right)=\frac{1}{1+e^{-2\zeta_{i}}}-1, \end{array}$$ (9)

The back-propagation method is a supervised training algorithm and it is commonly used for training MLF [25]. Mathematically training a network means minimizing the objective function *E*,

$$\begin{array}{c} E=\sum \frac{1}{2}{\left(x_{o}-{\hat{x}}_{o}\right)}^{2}, \end{array}$$ (10)

where *x*_*o*_ is the target stream flow and ${\hat{x}}_{o}$ is the computed value from the output neurons.

The work introduced is this paper aimed for high accuracy with no limits for training time. Therefore using multiple hidden layers is highly recommended [25]. The choice of number of neurons and layers depends mainly on the total number of hidden nodes of the whole neural network. In addition, a decision relating to the number of layers and proportion of neurons between the first and second hidden layer is required. In [26], some methods were used for determining the number of neurons in the hidden layers, such as the Rule of Thumb method which states the following:

- The number of hidden layer neurons are 2/3 of the size of the input layer neurons [27];
- The number of hidden layer neurons should be less than twice of the number of neurons in the input layer;
- The number of hidden neurons should be in the range between the size of the input layer and the output layer neurons.

However, the complexity of the activation function applied on the neurons represents an important impact on the network response. Therefore, the rule of thumb method may not be applicable for some applications. There is also the simple method, which takes the same number of nodes in the input, output and hidden node [28].

This work uses the most common method, the two-phase method, which mainly depends on trial and error. The data is divided into four groups, where two data groups are used in the first phase to train the network and one group is used to test the network in the second phase. The fourth group is used to predict the output values of the trained network. This method is repeated for different number of hidden layer’s neurons to get the best network performance [29].

**Fig 13:**
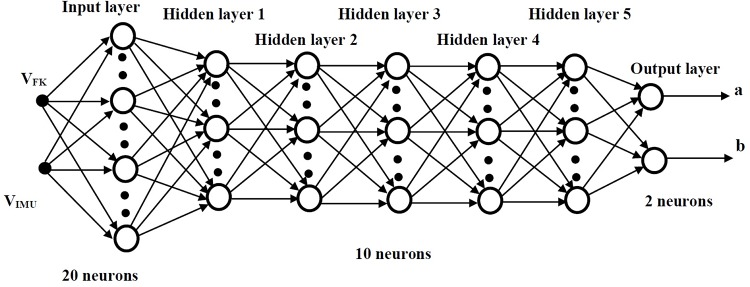
Overall MLF structure including hidden layers.

The proposed MLF consists of input and output layers, as shown in Fig 13, in addition to 5 hidden layers. The input layer has 20 neurons and the output layer consists of 2 neurons, while the hidden layers have 10 neurons each (20-10-10-10-10-10-2). This choice of such structure will be explained further in section 5

**Fig 14:**
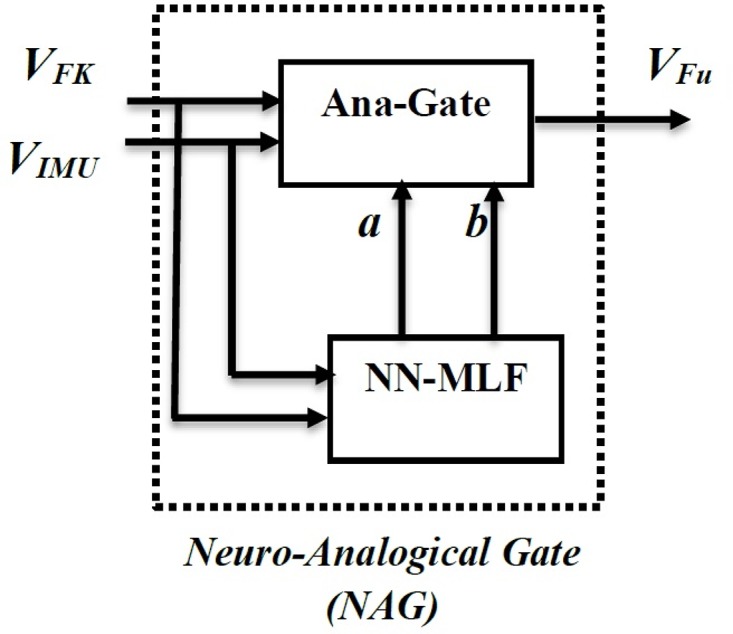
Neuro-analogical gate (NAG).

The neural network is trained using the inputs *V*_*FK*_ and *V*_*IMU*_ as input vectors and the *a* and *b* variables are the output vectors. Furthermore, the output vectors *a* and *b* values will be used to tune the analogical gate to the values of *V*_*Cam*_ as shown in Fig 14.

Choosing the proper structure for the neural network depends on many aspects; some of the them were described in the previous section. These structures were tested, and the decision was taken according to the mean square error value that resulted from the target and the estimated output values, as shown in Table 5.

**Table 5:** Mean square error for different MLF-NN structures.

Structure	Mean Square Error	Hidden Layers
20 − 10 − 10 − 2	1.391*e*^−27^	2
20 − 5 − 5 − 5 − 5 − 5 − 2	7.623*e*^−30^	5
10 − 5 − 5 − 5 − 5 − 5 − 2	5.539*e*^−28^	5
20 − 10 − 10 − 10 − 10 − 10 − 2	2.892*e*^−30^	5

**Fig 15:**
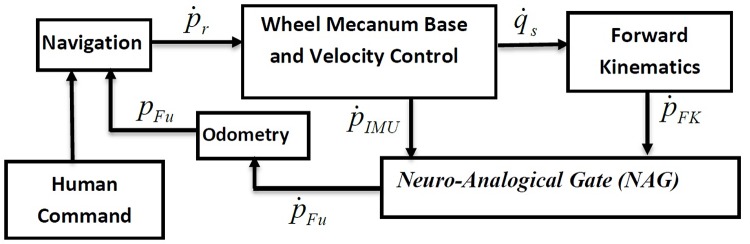
The apartment node map.

A conclusion about the structure can be generated using least mean square error, which is very important in our application, as high accuracy in the chair trajectory is required. The network presented in Fig 13 is trained using the sample data to find the weights and biases of the network. Then the network is used with in the NAG structure presented in Fig 14 and tested for another 25 samples. Finally, the NAG gate is used within the wheel chair control structure shown in Fig 15 and applied for the whole experiment.

The control structure shown in Fig 15 demonstrates the main objective of the NAG, where its main inputs are the velocity coordinates estimated from the forward kinematics (${\dot{P}}_{FK}$) analysis and the velocity coordinates from the IMU readings (${\dot{P}}_{IMU}$). The output is the velocity fused coordinates (${\dot{P}}_{Fu}$), which is fed directly to the odomerty algorithms to generate the fused trajectory (*P*_*Fu*_).

## 5 Experimental Results

**Fig 16:**
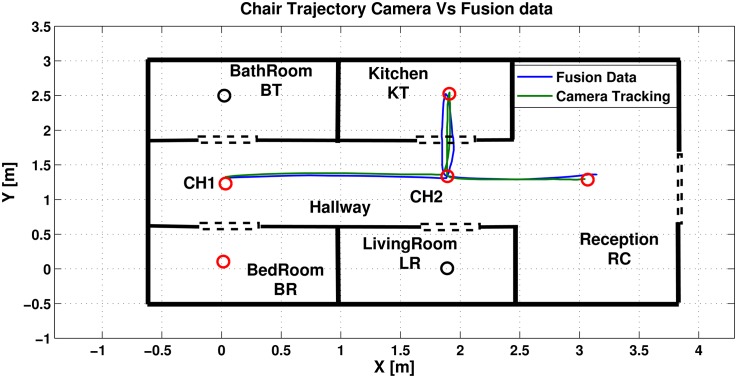
Wheel chair trajectories resulted from NAG and camera positioning systems.

This section presents the results of three main experiments to illustrate the performance of the NAG. Firstly, the experiment presented in section (2) is considered where Fig 16 shows the trajectory of the camera tracking system and the results from the NAG system.

The figure presents the camera system trajectory versus the fusion trajectory. It is quite noticeable that the new trajectory is much more aligned with the camera trajectory, with mean a error of 0.39[*m*]. There is a reduction of mean error with a percentage of 80% in comparison to FK and 70% in comparison to the IMU system.

**Fig 17:**
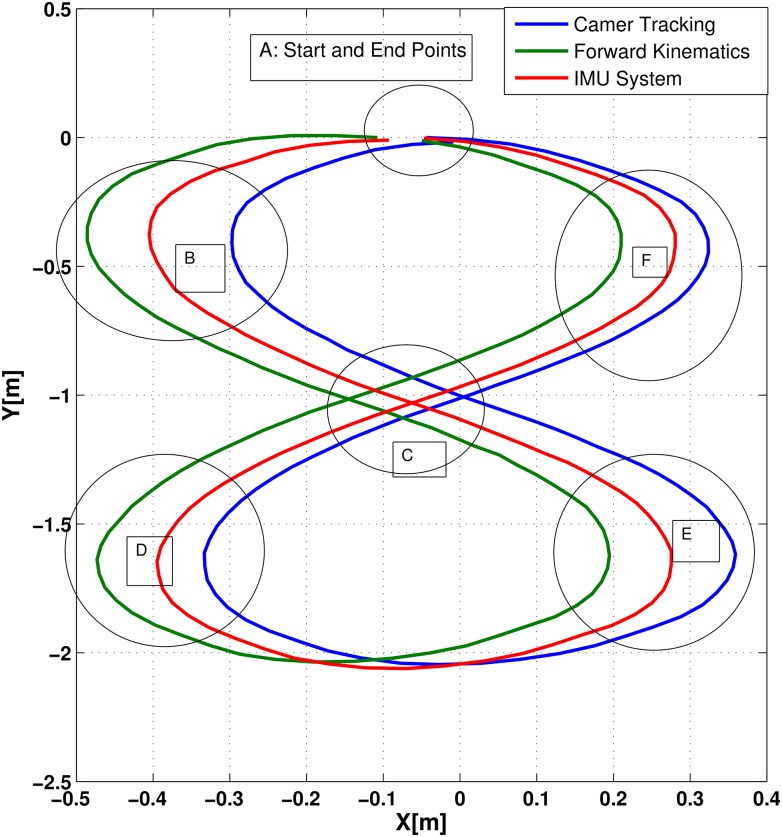
Infinity shape trajectories for FK, IMU and camera positioning systems.

The NAG fusion system was tested for infinity trajectory as well as shown in Fig 17 which represents the trajectories of the FK, IMU and camera positioning systems.

**Fig 18:**
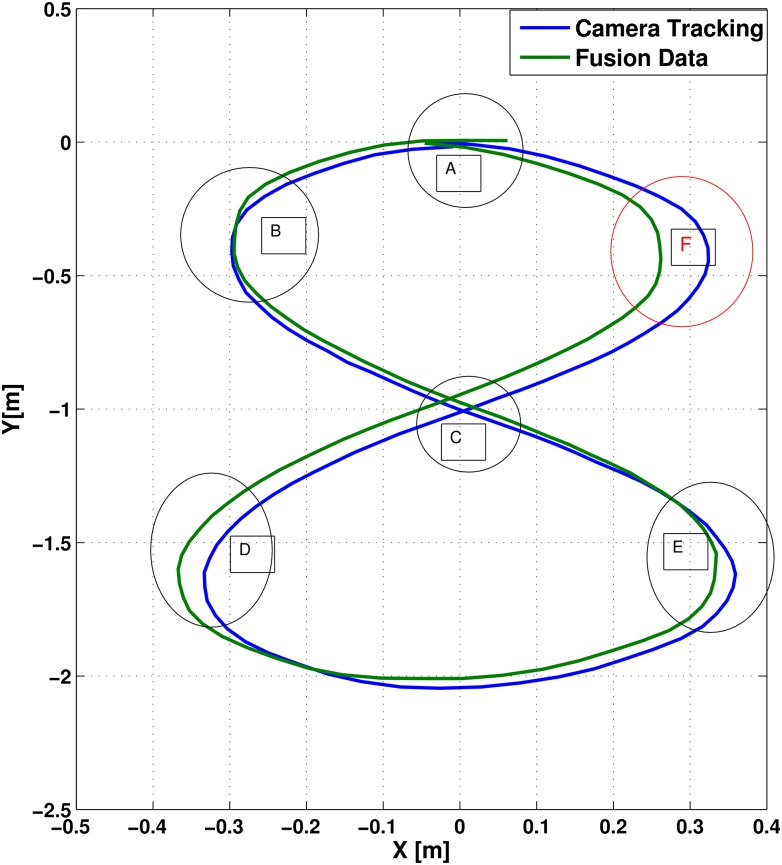
Infinity shape trajectories from NAG and camera positioning systems.

The figure shows the noticeable errors between the trajectories. After applying the proposed NAG fusion system, the trajectory presented in Fig 18 shows that the NAG generated trajectory is more accurate than the ones generated from the FK and IMU positioning systems.

In Fig 17, 6 main attractive zones can be observed:Zones A, B, C, D, E and F. Zone A: the start and end point, where the chair will start and ends its infinity shape trajectory. The data collected from the IMU and FK positioning systems show that they do not meet at the main point. The camera data shows the real performance where the chair ends at the same point that it started.

The curve zones (B, D, E and F) show the high diversity in trajectory readings the IMU and FK systems in comparison to the camera tracking systems. Such errors will cause false readings and affect the performance of the wheel chair navigational system. We can also notice at the intersection point C where the three trajectories intersections are not even close to each other.

After applying the proposed NAG fusion system, that the generated trajectory overcame the errors and diversities in the zones (B, D and E) as shown in the Fig 18. Zone A shows that the start and end points intersects again with much fewer errors than the ones generated from IMU and FK trajectories. However, zone F shows high errors in comparison to the other zones. This problem may be solved by increasing the data used for training, but for such procedures, the chair should train new network parameters after each operational trajectory with the camera trajectory system.

**Fig 19:**
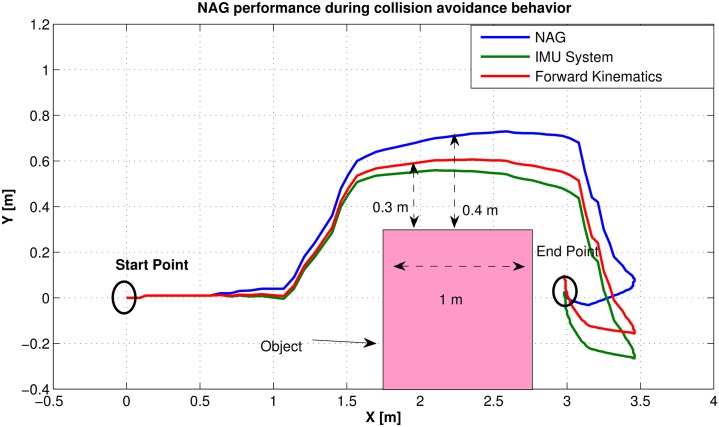
NAG performance during collision avoidance behavior.

The efficient performance of the proposed NAG system is elaborated in the following experiment. The main objective of the experiment to illustrate the performance of the chair when an unexpected object is introduced to the environment as shown in Fig 19. The wheel chair should drive from the starting point to the end point without colliding with any object. Therefore, eight ultrasonic sensors are attached to the platform and the algorithm of collision avoidance is introduced in [23]. The object width is one meter long as the wheel chair approaches the collision avoidance behavior is introduced to the system with priority higher than the position control system. After passing the object the position control will have priority on operation and drive towards the end point. The difference in performance between the three positioning systems is illustrated in the figure. The measurements accumulated errors found in the IMU and FK systems shows that the chair drives with distance of 0.3 meters far from the object, which is half the width of the wheel chair. Accordingly, the chair should be colliding with the object with these measurements. Alternatively, the trajectory representing the NAG shows its efficient performance in avoiding the object and reaching the end point, while the other systems trajectories shows distance errors at the end point.

## 6 Conclusion

A novel method was developed to fuse the data of two internal sensors (rotatory encoders and IMU) to match the measurements of an external camera tracking system. This fusion system generated the most accurate coordinates for a wheel chair for special needs users. The proposed method uses the means of neural networks to tune the parameters of analogical gate which fuses the internal sensor position data. The new method, Neuro-Analogical Gate (NAG), is used for two experimental data. the first to drive in straight lines within a known apartment environment and in infinity shape. The error difference between the NAG trajectory and the camera system is around 70%–80% less compared to the internal sensors. Such results prove the efficient performance of the system.

## Supporting Information

S1 File Tuned operating points. This file includes the operating points used to tune the analogical gates and to train the nueral network.
(XLSX)
